# Acute (14‐Day) and Subchronic (90‐Day) Toxicity Evaluation of the Dried Fruit Spice *Xylopia aethiopica* (Dunal) A. Rich. (Annonaceae) in Male and Female Wistar Rats

**DOI:** 10.1155/jt/2002718

**Published:** 2026-05-21

**Authors:** Merline Ymele Nguedia, Ornella Bernie Kami Nkuimi, Borelle Mafogang, Herve Herve Abaissou Ngatanko, Dieudonne Njamen, Stephane Zingue

**Affiliations:** ^1^ Department of Biological Sciences, Faculty of Science, University of Maroua, P.O. Box 814, Maroua, Cameroon, uni-maroua.citi.cm; ^2^ Department of Animal Biology and Physiology, Faculty of Science, University of Yaounde 1, P.O. Box 812, Yaounde, Cameroon, uy1.uninet.cm; ^3^ Department of Biochemistry, Faculty of Science, University of Yaoundé 1, P.O. Box 812, Yaounde, Cameroon, uy1.uninet.cm; ^4^ Department of Pharmacotoxicology and Pharmacokinetics, Faculty of Medicine and Biomedical Sciences, University of Yaounde 1, P.O. Box 1364, Yaounde, Cameroon, uy1.uninet.cm

**Keywords:** acute toxicity, spice, subchronic toxicity, Wistar rat, *Xylopia aethiopica*

## Abstract

**Background:**

*Xylopia aethiopica* (XAE) fruit (Ethiopian pepper) is a culinary spice widely used in African countries. It has numerous biological properties including potent anticancer properties; however, there is a lack of data on its safety, which justifies this study.

**Methods:**

Animals were administered a unique dose of 2000 mg/kg for the acute (14‐day) toxicity and three doses (75, 150, and 300 mg/kg BW) of the subchronic (90‐day) oral toxicity of ethanol dry fruit of XAE extract guideline numbers 423 and 408, respectively. Behavioral, morphological (body weight and relative weight of the organs), biochemical, hematological, and histological parameters of toxicological interest were evaluated with organs, such as the lung, liver, kidney, breast, and testes, just to name a few.

**Results:**

An LD_50_ > 2000 mg/kg was found after an acute exposure to XAE in young female rats. After 90 days of administration, XAE induced no significant changes in body weight and relative weights of the organs. In both female and male rats, no significant changes were observed in the tested biochemical parameters (creatinine, total proteins, triglycerides, total cholesterol, HDL, LDL, ALT, AST, and urea), except for the bilirubin levels, which slightly decreased in males at a dose of 300 mg/kg BW. No change was observed in the histological sections of organs of interest in toxicology (spleen, lungs, kidneys, liver, and heart) as well as in the reproductive organs (vagina, uterus, and ovary for females and testes, seminal vesicle, prostate, and epididymis for males). A significant decrease was observed in the level of hematological parameters, such as platelet levels in both female (*p* ≤ 0.001) and male (*p* ≤ 0.01) Wistar rats when compared to the control. This decrease in platelet concentration observed in experimental rats suggests that XAE may induce thrombocytopenia, possibly through an adverse effect on thrombopoiesis.

**Conclusion:**

Taken altogether, the observations therefore showed that long‐term administration of XAE dry fruit extract might induce thrombocytopenia and anemia, particularly at higher doses, warranting caution regarding long‐term use.

## 1. Introduction

The inclusion of food components in traditional dishes has demonstrated numerous health benefits, as evidenced by several studies [1, 2]. Consequently, the concept of functional foods has emerged, generating significant scientific interest in the utilization of spices in traditional cuisine. However, despite their historical use as both food and medicine, it would be simplistic to presume that plants are inherently nontoxic merely due to their “natural” status. The safety of medicinal herbs cannot be assumed solely based on their natural origin [3]. Moreover, the acute or chronic administration of plant‐based foods or herbs does not preclude the possibility of toxicity, even at low levels, especially if external signs of toxicity are not readily observed. It is noteworthy that many commonly consumed foods contain constituents that could be considered poisonous, such as oats, alpha gliadin in wheat and rye, glycosides in fruit seeds, cyanogenic thiocyanates in brassica vegetables, alkaloids in the Solanaceae family, and lectins in various pulses including soybeans and red kidney beans [4]. Therefore, studies investigating the formulation of traditional extracts are imperative to validate their usage, efficacy, and safety, as the toxicity of each substance, even if natural, is dosage‐dependent [5].

*Xylopia aethiopica (XAE),* commonly referred to as Ethiopian pepper, is a flowering plant classified as an angiosperm within the Annonaceae family, also known as the custard apple family, and is widely distributed in the evergreen rainforests of tropical and subtropical Africa [6]. It produces fragrant fruits [7] and has been traditionally used for treating various ailments including bronchitis, cough, rheumatism, muscular pain, headaches, malaria, dysentery, uterine fibroids, amenorrhea, boils, sores, wounds, and cancer [8, 9]. In Cameroon, the dry fruits of XAE are used in “Nkui,” a traditional dish frequently eaten by women, particularly in the final trimester of pregnancy to aid in childbirth and during the 3–5 months following childbirth to reduce the risk of postpartum hemorrhage [10]. Previous studies have highlighted the antimicrobial properties of XAE leaves and their potential to alleviate stomach damage in streptozotocin‐induced diabetic rats [11]. Studies on XAE‐treated rats have indicated hypokalemic and hypolipidemic effects, as well as minimal or no hepatotoxic effects [12, 13]. Moreover, the seed extract demonstrates hepatoprotective and antioxidant properties similar to those of Livolin forte [14]. Nonetheless, an increase in creatinine levels in rabbits has been observed following administration of the fruit extract of XAE [15]. In our ongoing search for cancer‐preventing functional foods, we have recently demonstrated that XAE prevents breast adenocarcinoma in female rats [16].

The XAE fruit comprises components that may collectively enhance the nutritional value of other food substances and contains significant levels of certain minerals that aid in enzyme catalysis, the maintenance of homeostasis, and support immune function [17]. Using label‐free proteomics, a total of 677 proteins were identified in XAE seeds, with 114 proteins common between the samples from Nigeria and Ghana, among which 48 were significantly changed [18]. Using HPLC fingerprint evaluation, the similarities of the samples from Benin, Cameroon, Nigeria, and Ghana were established. The samples from Cameroon were found to have the highest average content of xylopic acid, while those from Benin had the lowest average content [19]. This fruit was found to contain six cinnamoylquinic acid derivatives and twenty‐four flavonoid glycosides with chrysoeriol‐7‐*O*‐glycosides being the main constituents after HPLC‐DAD‐ESI (Ion Trap)‐MS^n^ and UPLC‐ESI‐QTOF‐MS^2^ analysis [20]. Phytochemical screening showed the presence of alkaloids, tannins, flavonoids, glycosides, saponins, balsam, cardiac glycoside, volatile oil, and steroids in this extract, accompanied by moderate antioxidant activities [21].

Despite its widespread use as a spice in traditional medicine and nutrition, there is a need to examine its effects on different organs to inform consumers of possible consequences. While this study adhered to prescribed guidelines and regulations, no reports on the safety evaluation of XAE have been published. Therefore, this study aims to evaluate the acute (24 h) and subchronic (90 days) oral toxicities of XAE dry fruit ethanol extract in Wistar rats.

## 2. Materials and Methods

### 2.1. Plant Material and Its Preparation

XAE dry fruits were collected in Bafut village (Northwest Cameroon) on December 27, 2021, at coordinates N 6°5′33.07758″ (latitude) and E 10°6′33.3159″ (longitude). Authentication was conducted by Dr. Eric T. NGANSOP at the National Herbarium of Cameroon in Yaounde, where a reference specimen, No. 55011/HNC, was used for comparison, and a sample was deposited.

The dry fruits were ground into powder form (500 g) and then macerated in 5 L of 95% ethanol for 72 h at 25°C. Filtration was performed using a fine sieve and finally with a Whatman No. 4 filter paper. The filtrate underwent concentration through a rotary evaporator under reduced pressure of 180 mbar at 40°C, yielding 50 g of crude extract with a 10% yield. The extract was conserved in sealed plain bottles, labeled, and kept at 4°C. Doses were prepared fresh by dissolving the required quantity of extract in distilled water to the indicated concentrations, four times a week.

### 2.2. Care and Selection of Animals

Both sexes of healthy rats were used and treated according to the European Union on Animal Care (CEE Council 86/609) guidelines adopted by the Cameroon Institutional National Ethics Committee. As recommended by the OECD Guideline No. 423, female Wistar rats aged 8–10 weeks old, weighing 120–150 g, were used for acute toxicity (14 days), while subchronic toxicity (90 days) included both males and females aged 6–7 weeks old, weighing 100–120 g. The rats were supplied and accommodated in plastic cages in the laboratory of Animal Physiology and Pharmacology at the University of Maroua for 2 weeks before the start of the experiment. They were separated by sex to avoid mating, fed, and provided with free access to clean tap water *ad libitum*. The animals were subjected to an ambient temperature and a light/dark cycle of 12 h each.

### 2.3. Assessment of Acute Oral Toxicity

XAE oral acute toxicity administration was evaluated in rats according to the OECD protocol, Guideline No. 423 for the testing of chemical substances (method by class of acute toxicity) adopted on December 17, 2001 [22]. Before the experiment began, animals were divided into two groups, each comprising three female rats. They were weighed and identified with markings. Before substance administration, they underwent fasting overnight, excluding water. The control group received distilled water, while the test group was given the extract at a single dose of 2000 mg/kg BW. After administration, food was withheld for a further 4 h, after which they were followed up for observation at 30 min, 1, 2, 3, and 4 h to record any behavioral changes (fur, eyes, respiration, behavior pattern, diarrhea, and tremors) and toxicity. In addition, daily observations were conducted for 14 days after the administration of the substances. A 2‐week test was later repeated following the same protocol as described above for verification. Animals were weighed three times per week; no moribund animals or those showing severe distress or signs of severe pain were recorded during the experiment.

### 2.4. Assessment of Subchronic Oral Toxicity

This experiment was done according to the Guideline No. 408 of the OECD [23]. To accomplish this, 72 animals aged 6–7 weeks, weighing 100–120 g, were subdivided into 6 groups, each consisting of 12 rats (6 males and 6 females).

Control Group 1 was given distilled water, whereas the test groups received the extract orally by gavage on a daily basis for 90 days at doses with anticancer effects (75, 150, and 300 mg/kg). Groups 5 and 6, each consisting of 12 rats (6 females and 6 males), served as the satellite control group and the satellite group, respectively, receiving the highest dose of the extract (300 mg/kg). On the 90^th^ day, all test groups and control groups (Groups 1–4) underwent a 12‐h water‐free fast. The satellite groups (Groups 5–6) were later observed for an additional 28 days without treatment to evaluate the persistence, aggravation, or reversibility of probable toxic effects.

During the treatment period, behavioral parameters and weight changes were assessed weekly. Subsequently, the animals were euthanized by decapitation following anesthesia induced by diazepam (10 mg/kg) and ketamine (50 mg/kg). Arterio‐venous blood was collected for hematological analysis (in EDTA tubes for complete blood count) and biochemical analysis (in serum tube or plain tube). Organs, such as the liver, kidneys, lungs, mammary gland (for females), and prostate and testes (for males), as well as other organs including the heart, spleen, stomach, ovaries, vagina, uterus, and seminal vesicle, were removed, weighed, and fixed in 10% formaldehyde for histopathological analyses and determination of relative weights, respectively.

### 2.5. Hematological Analysis

Blood drawn via cardiac puncture into EDTA tubes was promptly utilized to assess various parameters including mean corpuscular volume (MCV), hemoglobin (Hb), mean corpuscular hemoglobin (MCH), red blood cell (RBC) count, mean corpuscular hemoglobin concentration (MCHC), total white blood cell (WBC) count, and differential WBC count (lymphocytes and granulocytes), as well as platelet count. These assessments were conducted using a HumaCount 30TS Automated Hematology Analyzer (HUMAN Diagnostics Worldwide, Wiesbaden, Germany).

### 2.6. Biochemical Analysis

The serum, obtained by centrifuging blood collected in plain tubes (serum tubes) at 3000 rpm at −5°C for 15 min, was preserved at −20°C until further processing. Parameters, such as high‐density lipoprotein (HDL‐C), total cholesterol (TC), triglycerides (TG), aspartate transaminase (AST), alanine transaminase (ALT), bilirubin, urea, and creatinine, were analyzed using reagent kits from Fortress Diagnostics Limited (Muckamore, United Kingdom). Total proteins were measured using the Biuret reagent [24].

### 2.7. Histopathological Analysis

The liver, lung, kidney, ovaries, uterus, vagina, epididymis, and testes were carefully removed and weighed. The organs were preserved in a 10% buffered formalin solution and subsequently embedded in paraffin to create thin sections (5 μm) that were stained with hematoxylin and eosin. These sections were then examined under a Zeiss Axioskop 40 microscope fitted with an AxioCam‐MRc digital camera connected to a computer. Image analysis was performed using Carl Zeiss AxioCam MR—MRGrab1.1 and AxioVision 3.1 software (Hallbergmoos, Germany).

### 2.8. Statistical Analysis

The results are displayed as the mean ± standard error of the mean (SEM). Statistical evaluation for both male and female participants encompassed both one‐way and two‐way analysis of variance (ANOVA), succeeded by Tukey’s multiple comparison tests via GraphPad Prism software Version 8.01. Significance was attributed to *p*‐values below 0.05 when compared to the control group.

## 3. Results

### 3.1. Acute (24‐h) Toxicity Test

There were no notable alterations in body weight observed over the 14‐day monitoring period (as depicted in Figure 1(a)). During the 14‐day observation period (Supporting data (available here)), there were no fatalities or indications of toxicity noted in the behavioral assessments (including mobility, sensitivity to noise, fur condition, grooming behavior, and aggressiveness) of the treated rats. This suggests that the LD_50_ value exceeds 2000 mg/kg.

FIGURE 1Body weight assessment of female rats after a unique oral administration at the dose of 2000 mg/kg of ethanol extract of *Xylopia aethiopica* (XAE) dry fruits (a) and body weight evaluation in female (b) and male (c) rats (*n* = 5) orally administered for 90 days. Control rats received distilled water (vehicle); XAE = rats treated with the ethanolic extract of *X. aethiopica* dry fruits at the respective doses of 75, 150, and 300 mg/kg BW. Control‐SAT: satellite control treated with vehicle; XAE 300‐SAT: satellite of high dose of extract (300 mg/kg). Data are expressed as mean ± SEM (*n* = 5). No significant change observed.

**Figure:**
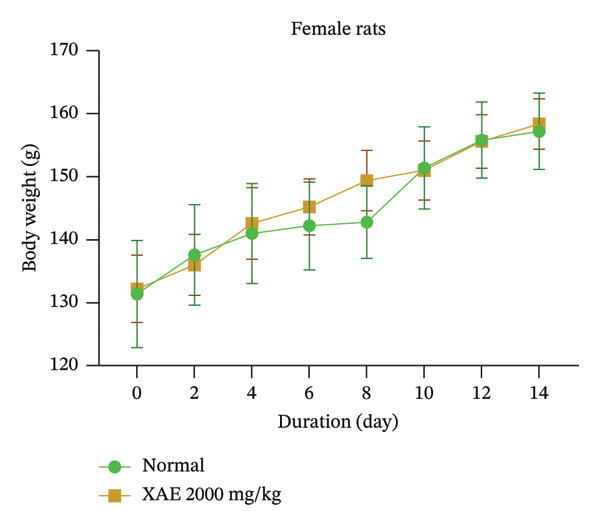
(a)

**Figure:**
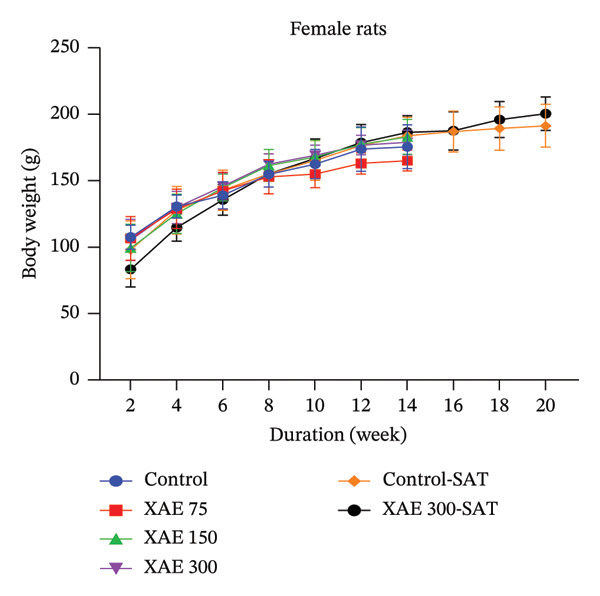
(b)

**Figure:**
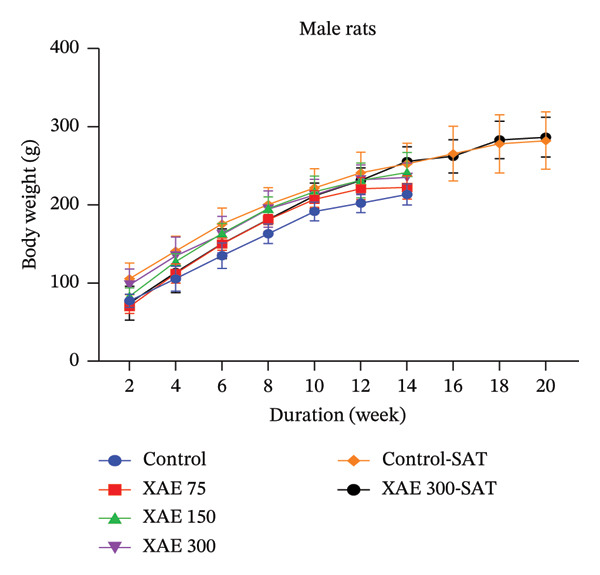
(c)

### 3.2. Subchronic (90‐day) Toxicity Test

#### 3.2.1. Body Weight

In the course of the experimental period, there were no deaths in the groups treated with XAE or in the control groups. In the same line, no significant changes were observed in the body weights in both female (Figure 1(b)) and male (Figure 1(c)) rats.

#### 3.2.2. Relative Weights of Organs

No significant changes were observed in the relative weights of both female (Table 1) and male (Table 2) rats after 90 days of continuous treatment with ethanol dry fruit extract of XAE.

**TABLE 1:** Relative weight of organs as well as vagina and uterine epithelial heights of female Wistar rats submitted to a 90‐day oral treatment with XAE.

Parameters	Female rat					
	Control	XAE 75	XAE 150	XAE 300	Control‐SAT	XAE 300‐SAT
*Relative weight of organs (g/100 g BW)*						
Liver	4.68 ± 0.18	4.51 ± 0.075	5.04 ± 0.39	4.95 ± 0.05	4.72 ± 0.25	5.66 ± 0.22
Stomach	1.48 ± 0.15	1.56 ± 0.10	1.56 ± 0.11	1.79 ± 0.84	1.82 ± 0.18	1.65 ± 0.06
Kidneys	0.87 ± 0.06	0.88 ± 0.04	0.89 ± 0.12	0.96 ± 0.43	0.89 ± 0.06	1.10 ± 0.06
Lungs	1.05 ± 0.06	1.14 ± 0.08	1.33 ± 0.17	1.09 ± 0.25	0.79 ± 0.20	1.35 ± 0.09
Spleen	0.51 ± 0.04	0.51 ± 0.04	0.68 ± 0.04	0.55 ± 0.02	0.74 ± 0.10	0.57 ± 0.03
Heart	0.56 ± 0.03	0.55 ± 0.02	0.70 ± 0.01	0.60 ± 0.02	0.53 ± 0.02	0.61 ± 0.04
Ovaries	0.15 ± 0.03	0.14 ± 0.01	0.14 ± 0.01	0.14 ± 0.02	0.14 ± 0.01	0.16 ± 0.01
Uterus	0.11 ± 0.01	0.11 ± 0.02	0.11 ± 0.01	0.10 ± 0.01	0.10 ± 0.01	0.09 ± 0.01

*Epithelial height (μm)*						
Uterus	7.54 ± 0.51	8.13 ± 0.43	7.14 ± 0.65	7.53 ± 0.83	6.68 ± 0.60	6.52 ± 0.39
Vagina	21.50 ± 2.30	21.10 ± 2.60	20.20 ± 1.70	19.90 ± 2.00	20.30 ± 1.50	20.70 ± 1.80

*Note:* Control rats received distilled water (vehicle); XAE = rats treated with the ethanolic extract of *X. aethiopica* dry fruits at the respective doses of 75, 150, and 300 mg/kg BW. Control‐SAT: satellite control treated with vehicle; XAE 300‐SAT: satellite of high dose of extract (300 mg/kg). Data are expressed as mean ± SEM (*n* = 6).

**TABLE 2:** Relative weight of organs in male Wistar rats submitted to a 90‐day oral treatment with XAE.

Organs (g/100 g BW)	Male rat					
	Control	XAE 75	XAE 150	XAE 300	Control‐SAT	XAE 300‐SAT
Liver	6.10 ± 0.19	6.82 ± 0.36	6.68 ± 0.26	6.65 ± 0.24	6.81 ± 0.61	7.59 ± 0.24
Stomach	1.56 ± 0.10	1.60 ± 0.55	1.79 ± 0.11	1.67 ± 0.08	1.67 ± 0.04	1.89 ± 0.01
Kidneys	1.18 ± 0.05	1.35 ± 0.05	1.26 ± 0.08	1.17 ± 0.06	1.51 ± 0.15	1.51 ± 0.06
Lungs	1.34 ± 0.17	1.40 ± 0.09	1.37 ± 0.17	1.39 ± 0.16	1.34 ± 0.15	1.49 ± 0.13
Spleen	0.71 ± 0.02	0.57 ± 0.07	0.61 ± 0.02	0.59 ± 0.05	0.65 ± 0.04	0.66 ± 0.03
Heart	0.67 ± 0.04	0.74 ± 0.04	0.81 ± 0.05	0.71 ± 0.04	0.76 ± 0.04	0.83 ± 0.07
Testes	2.33 ± 0.04	2.64 ± 0.06	2.58 ± 0.26	2.62 ± 0.11	2.62 ± 0.14	2.58 ± 0.09
S. vesicle	0.83 ± 0.11	1.26 ± 0.14	1.31 ± 0.14	1.28 ± 0.16	0.96 ± 0.11	1.15 ± 0.08
Epididymis	0.86 ± 0.02	0.95 ± 0.03	0.94 ± 0.07	0.96 ± 0.07	1.08 ± 0.04	0.93 ± 0.08

*Note:* Control rats received distilled water (vehicle); XAE = rats treated with the ethanolic extract of *X. aethiopica* dry fruits at the respective doses of 75, 150, and 300 mg/kg BW. Control‐SAT: satellite control treated with vehicle; XAE 300‐SAT: satellite of high dose of extract (300 mg/kg). Data are expressed as mean ± SEM (*n* = 6).

#### 3.2.3. Hematological Parameters

Tables 3 and 4 illustrate the impact of XAE on hematological parameters for female and male rats, respectively. There were no notable changes observed in WBC counts in either female or male rats.

**TABLE 3:** Hematological parameters in female Wistar rats submitted to a 90‐day oral treatment with XAE.

Parameters	Female rats					
	Control	XAE 75	XAE 150	XAE 300	Control‐SAT	XAE 300‐SAT
WBC (10^3^/μL)	1.68 ± 0.39	2.26 ± 0.34	0.58 ± 0.02	0.98 ± 0.11	1.74 ± 0.50	1.17 ± 0.57
LYM%	90.6 ± 0.47	67.90 ± 2.51^∗∗∗^	82.90 ± 3.96	85.9 ± 1.45	72.40 ± 4.77	75.20 ± 2.24
GRAN%	3.43 ± 0.16	14.60 ± 0.82^∗∗∗^	8.00 ± 1.09^∗∗∗^	7.70 ± 0.77^∗∗∗^	8.40 ± 3.60	7.70 ± 2.14
RBC (10^6^/μL)	3.76 ± 0.12	0.884 ± 0.24^∗∗∗^	3.56 ± 0.20	3.72 ± 0.15	3.78 ± 0.37	3.51 ± 0.72
Hemoglobin (g/dL)	9.53 ± 0.88	3.24 ± 0.55^∗∗∗^	7.02 ± 0.94	7.30 ± 0.41	7.00 ± 1.53	7.70 ± 0.71
Hematocrit (%)	26.50 ± 0.70	7.00 ± 1.00^∗∗∗^	19.60 ± 0.74	19.90 ± 0.26	23.70 ± 2.40	26.90 ± 3.80
MCV (fL)	52.90 ± 3.40	49.20 ± 1.70	54.80 ± 1.60	53.60 ± 1.01	56.50 ± 0.63	56.90 ± 1.02
MCH (pg)	20.60 ± 0.99	21.30 ± 1.49	19.70 ± 0.23	19.70 ± 0.19	19.40 ± 0.47	21.50 ± 1.10
MCHC (g/dL)	353.00 ± 2.02	336.0 ± 3.57	354.00 ± 7.55	359.00 ± 2.92	345.00 ± 5.54	351.00 ± 6.68
Platelets (10^3^/μL)	375.00 ± 95.20	170.00 ± 30.70^∗∗∗^	166.00 ± 16.20^∗∗∗^	206.00 ± 24.00^∗∗∗^	286.00 ± 67.70	339.00 ± 81.20

*Note:* Control rats received distilled water (vehicle); XAE = rats treated with the ethanolic extract of *X. aethiopica* dry fruits at the respective doses of 75, 150, and 300 mg/kg BW. Control‐SAT: satellite control treated with vehicle; XAE 300‐SAT: satellite of high dose of extract (300 mg/kg). Data are expressed as mean ± SEM (*n* = 5). Significance against the control group: ^∗∗∗^ *p* < 0.001. LYM% = % lymphocyte, GRAN% = % granulocyte.

Abbreviations: MCH = mean corpuscular hemoglobin, MCHC = mean corpuscular hemoglobin concentration, MCV = mean corpuscular volume, RBC = red blood cell, WBC = white blood cell.

**TABLE 4:** Hematological parameters in male Wistar rats submitted to a 90‐day oral treatment with XAE.

Parameters	Male rats					
	Control	XAE 75	XAE 150	XAE 300	Control‐SAT	XAE 300‐SAT
WBC (10^3^/μL)	1.58 ± 0.46	1.58 ± 0.22	1.66 ± 0.32	1.22 ± 0.16	1.60 ± 0.55	1.66 ± 1.39
LYM%	91.60 ± 0.86	90.4 ± 0.56	87.50 ± 3.66	76.50 ± 2.46^∗∗^	91.90 ± 2.41	88.90 ± 2.17
GRAN%	6.30 ± 0.32	5.40 ± 0.21	5.88 ± 0.79	15.90 ± 1.02^∗∗∗^	8.51 ± 1.33	8.90 ± 1.34
RBC (10^6^/μL)	6.27 ± 0.21	6.89 ± 0.09	5.88 ± 0.28	4.88 ± 0.147^∗∗^	7.77 ± 0.21	7.73 ± 0.26
Hemoglobin (g/dL)	11.20 ± 1.00	13.60 ± 0.31	10.80 ± 0.97	8.94 ± 0.65	14.60 ± 0.44	14.90 ± 0.37
Hematocrit (%)	34.40 ± 1.30	38.80 ± 0.78	28.70 ± 1.89	26.60 ± 0.99^∗∗^	42.00 ± 1.12	40.70 ± 1.54
MCV (fL)	51.60 ± 2.59	55.90 ± 1.21	54.50 ± 0.63	54.50 ± 0.66	54.10 ± 0.91	52.60 ± 0.65
MCH (pg)	19.5 ± 0.33	19.70 ± 0.21	20.30 ± 0.15	19.40 ± 0.25	18.80 ± 0.17	19.30 ± 0.28
MCHC (g/dL)	355.00 ± 5.53	351.00 ± 6.38	373.00 ± 3.05	357.00 ± 8.29	347.00 ± 6.25	363.00 ± 3.23
Platelets (10^3^/μL)	786.00 ± 32.40	630.00 ± 57.00	797.00 ± 21.90	466.00 ± 3.33^∗∗^	697.00 ± 72.00	676.00 ± 60.50

*Note:* Control rats received distilled water (vehicle); XAE = rats treated with the ethanolic extract of *X. aethiopica* dry fruits at the respective doses of 75, 150, and 300 mg/kg BW. Control‐SAT: satellite control treated with vehicle; XAE 300‐SAT: satellite of high dose of extract (300 mg/kg). Data are expressed as mean ± SEM (*n* = 5). Significance against the control group: ^∗∗^ *p* < 0.01 and ^∗∗∗^ *p* < 0.001. LYM% = % lymphocyte, GRAN% = % granulocyte.

Abbreviations: MCH = mean corpuscular hemoglobin, MCHC = mean corpuscular hemoglobin concentration, MCV = mean corpuscular volume, RBC = red blood cell, WBC = white blood cell.

In female rats, a significant decrease (*p* < 0.001) was observed in lymphocyte % at the dose of 75 mg/kg, while granulocyte % exhibited a significant increase (*p* < 0.01) compared to the control. Additionally, RBC, Hb, and hematocrit significantly decreased (*p* < 0.001) in the XAE‐treated animals at a dose of 75 mg/kg compared to the control group. MCV, MCH, and MCHC levels showed no significant changes with XAE at different doses after 90 days and 28 days of observation. However, platelet counts were significantly decreased (*p* < 0.001) at all tested doses after 90 days of treatment (as shown in Table 3).

In male rats, the parameters lymphocyte %, RBC, hematocrit, and platelets exhibited significant decreases with XAE at a dose of 300 mg/kg, while granulocyte % increased significantly (*p* < 0.001) compared to the control group after 90 days of treatment. MCV, MCH, and MCHC showed no significant changes after 90 days of treatment and 28 days of observation (as depicted in Table 4).

After 28 days of observation, no significant changes were noted in the XAE 300‐SAT as compared to control‐SAT in both male and female rats.

#### 3.2.4. Biochemical Parameters

The impact of subchronic oral administration of XAE on biochemical parameters following 90 days of treatment and 28 days of observation in both female (depicted in Table 5) and male (illustrated in Table 6) rats is presented. In both female and male rats, no significant changes were observed in the tested biochemical parameters (creatinine, total proteins, TG, TC, HDL, LDL, ALT, AST, and urea) compared to the sex‐matched control rats after 90 days of treatment and 28 days of observation. However, bilirubin levels exhibited a slight decrease (*p* < 0.05) in the male rats treated with XAE at 300 mg/kg compared to the control rats. No significant changes were observed between the control/SAT rats and the SAT 300 mg/kg rats.

**TABLE 5:** Biochemical parameters in female Wistar rats submitted to a 90‐day oral treatment with XAE extract at the doses.

Parameters	Female rats					
	Control	XAE 75	XAE 150	XAE 300	Control‐SAT	XAE 300‐SAT
Protein	0.07 ± 0.01	0.06 ± 0.01	0.07 ± 0.01	0.07 ± 0.01	0.07 ± 0.01	0.08 ± 0.01
Bilirubin	0.16 ± 0.012	0.14 ± 0.02	0.15 ± 0.01	0.17 ± 0.02	0.19 ± 0.03	0.15 ± 0.02
Triglycerides	0.92 ± 0.02	0.94 ± 0.03	0.91 ± 0.02	0.90 ± 0.04	0.93 ± 0.02	0.94 ± 0.04
T. cholesterol	3.56 ± 0.24	3.47 ± 0.12	3.33 ± 0.13	3.64 ± 0.16	3.40 ± 0.09	3.62 ± 0.23
HLD	2.9 ± 0.26	2.69 ± 0.18	2.46 ± 0.14	2.88 ± 0.08	2.66 ± 0.19	2.55 ± 0.09
LDL (g/L)	0.94 ± 0.01	0.97 ± 0.01	0.93 ± 0.01	0.93 ± 0.01	0.90 ± 0.01	0.86 ± 0.04
ALAT	4.92 ± 0.12	5.01 ± 0.26	4.78 ± 0.23	5.14 ± 0.32	4.76 ± 0.14	5.47 ± 0.42
ASAT	5.39 ± 0.40	7.25 ± 0.38	6.33 ± 0.51	7.06 ± 0.32	7.68 ± 0.62	7.54 ± 0.53
Creatinine	0.5 ± 0.01	0.65 ± 0.03	0.54 ± 0.04	0.37 ± 0.05	0.47 ± 0.00	0.47 ± 0.06
UREA	0.06 ± 0.01	0.07 ± 0.01	0.07 ± 0.01	0.07 ± 0.01	0.07 ± 0.01	0.07 ± 0.01

*Note:* Control rats received distilled water (vehicle); XAE = rats treated with the ethanolic extract of *X. aethiopica* dry fruits at the respective doses of 75, 150, and 300 mg/kg BW. Control‐SAT: satellite control treated with vehicle; XAE 300‐SAT: satellite of high dose of extract (300 mg/kg). Data are expressed as mean ± SEM (*n* = 6).

**TABLE 6:** Biochemical parameters in male Wistar rats submitted to a 90‐day oral treatment with XAE extract at the doses of 75, 150, and 300 mg/kg.

Parameters	Male rats					
	Control	XAE 75	XAE 150	XAE 300	Control‐SAT M	SAT 300 M
Protein	0.06 ± 0.01	0.06 ± 0.01	0.06 ± 0.01	0.06 ± 0.01	0.06 ± 0.01	0.06 ± 0.01
Bilirubin	0.19 ± 0.02	0.19 ± 0.01	0.16 ± 0.02	0.10 ± 0.02^∗^	0.11 ± 0.02	0.09 ± 0.03
Triglycerides	1.11 ± 0.04	1.18 ± 0.07	1.13 ± 0.02	1.12 ± 0.04	1.12 ± 0.04	1.19 ± 0.07
T. cholesterol	3.33 ± 0.07	3.20 ± 0.04	3.24 ± 0.10	3.41 ± 0.15	3.23 ± 0.06	3.28 ± 0.03
HLD (g/L)	3.29 ± 0.17	3.56 ± 0.07	3.56 ± 0.11	2.88 ± 0.08	2.65 ± 0.08	2.79 ± 0.13
LDL (g/L)	1.13 ± 0.04	1.06 ± 0.01	1.10 ± 0.02	1.07 ± 0.03	1.06 ± 0.014	1.08 ± 0.04
ALAT	5.17 ± 0.58	4.92 ± 0.49	4.99 ± 0.16	6.25 ± 0.12	6.18 ± 0.59	5.65 ± 0.41
ASAT	8.93 ± 0.31	7.29 ± 0.56	7.64 ± 0.30	9.03 ± 0.49	8.35 ± 0.25	8.53 ± 0.49
Creatinine	0.5 ± 0.01	0.2 ± 0.01	0.2 ± 0.01	0.3 ± 0.01	0.5 ± 0.01	0.5 ± 0.1
UREA	0.08 ± 0.01	0.07 ± 0.01	0.07 ± 0.01	0.08 ± 0.01	0.08 ± 0.01	0.06 ± 0.01

*Note:* Control rats received distilled water (vehicle); XAE = rats treated with the ethanolic extract of *X. aethiopica* dry fruits at the respective doses of 75, 150, and 300 mg/kg BW. Control‐SAT: satellite control treated with vehicle; XAE 300‐SAT: satellite of high dose of extract (300 mg/kg). Data are expressed as mean ± SEM (*n* = 6). Significance against the control group ^∗^ *p* < 0.01, ^∗∗∗^ *p* < 0.001.

#### 3.2.5. Histopathological Analysis

**FIGURE 2:**
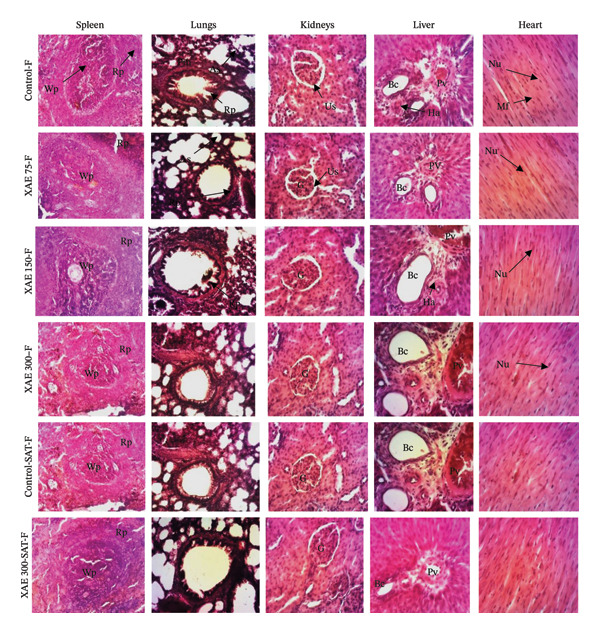
Effect of ethanol extract of *X. aethiopica* on the structure of the liver, lungs, kidney, spleen, and heart of female Wistar rats (HE, 100x). Control rats received distilled water (vehicle); XAE = rats treated with the ethanolic extract of *X. aethiopica* dry fruits at the respective doses of 75, 150, and 300 mg/kg BW. Control‐SAT: satellite control treated with vehicle; XAE 300‐SAT: satellite of high dose of extract (300 mg/kg). Data are expressed as mean ± SEM (*n* = 5). Liver: Vp = portal vein; He = hepatocyte; Bc = biliary canaliculus; Kidney: G = glomerulus; US = urinary space; Lung: Mw = muscular wall; As = alveolar sac; Pe = pulmonary epithelium; Spleen: Gc = germinal center; Wp = white pulp; Rp = red pulp; Heart: Mf = muscular fiber; N = nucleus.

**FIGURE 3:**
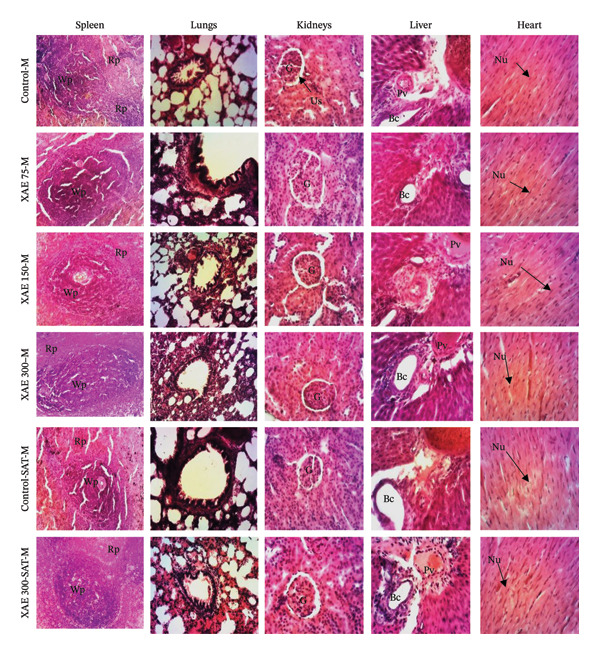
Effect of ethanol extract of *X. aethiopica* on the structure of the liver, lungs, kidney, spleen, and heart of male Wistar rats (HE, 100x). Control rats received distilled water (vehicle); XAE = rats treated with the ethanolic extract of *X. aethiopica* dry fruits at the respective doses of 75, 150, and 300 mg/kg BW. Control‐SAT: satellite control treated with vehicle; XAE 300‐SAT: satellite of high dose of extract (300 mg/kg). Data are expressed as mean ± SEM (*n* = 5). Liver: Vp = portal vein; He = hepatocyte; Bc = biliary canaliculus; Kidney: G = glomerulus; US = urinary space; Lung: Mw = muscular wall; As = alveolar sac; Pe = pulmonary epithelium; Spleen: Gc = germinal center; Wp = white pulp; Rp = red pulp; Heart: Mf = muscular fiber; N = nucleus.

**FIGURE 4:**
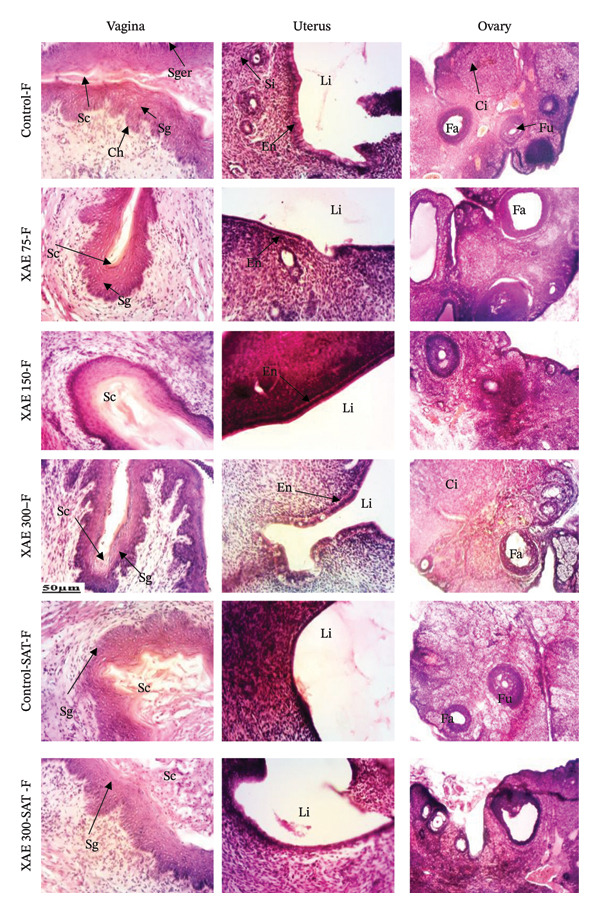
Effect of ethanol extract of *X. aethiopica* on the structure of the vagina, uterus, and ovaries of female Wistar rats (HE 100x). Control rats received distilled water (vehicle); XAE = rats treated with the ethanolic extract of *X. aethiopica* dry fruits at the respective doses of 75, 150, and 300 mg/kg BW. Control‐SAT: satellite control treated with vehicle; XAE 300‐SAT: satellite of high dose of extract (300 mg/kg). Data are expressed as mean ± SEM (*n* = 5). Sge = *Stratum germinativum*; Sg = *Stratum granulosum*, Sc = *Stratum corneum*, Lv = vaginal lumen, Ch = chorion Lu = lumen; Sc = stratum corneum; SG = stratum granulosum; Sg = stratum germinativum, Ch = chorion, testis; Gc = germ cells; Se = seminiferous epithelium, It = interstitial tissue, Lst = lumen of seminiferous tube; epididymis; Ee = epididymal epithelium, Spz = spermatozoon; seminal vesicle; Ct = connective tissue; Es = eosinophilic secretion. Ductus deferens; Mu = muscularis, Dde = ductus deferens epithelium, L = lumen.

The impact of XAE on the microarchitecture of the heart, spleen, liver, kidney, and lung in both female and male rats is presented in Figures 2 and 3, respectively. Histological examination of liver sections in both the control group and all treated groups revealed normal liver parenchyma architecture, characterized by a clear centrilobular vein and hepatocytes. The kidney exhibited normal parenchyma with a discernible glomerulus and urinary space. Across all treated groups, the lung displayed clear pulmonary epithelium, muscle wall, and alveolar sacs, while the spleen exhibited distinct white and red pulp. In all treated groups, the heart showed normal myocardial structure with distinct muscle fibers and myocyte nuclei. The control and treated groups showed typical uterine parenchyma with stroma, endometrium, and lumen as well as a normal vaginal parenchyma characterized by a germinal layer, a granular layer, and a horny layer (Figure 4).

**FIGURE 5:**
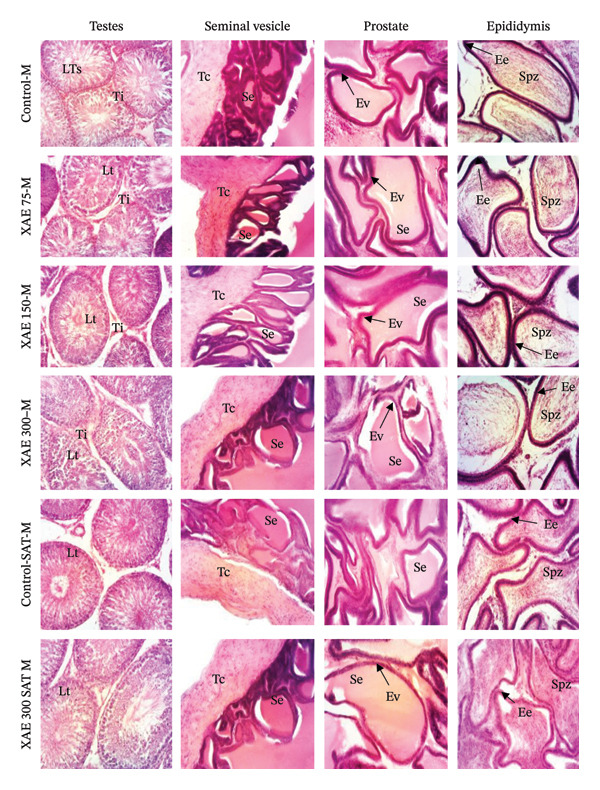
Effect of ethanol extract of *X. aethiopica* on the structure of epididymis, testis, vas deferens, prostate, and seminal vesicle of male (HE, 100x). Control rats received distilled water (vehicle); XAE = rats treated with the ethanolic extract of *X. aethiopica* dry fruits at the respective doses of 75, 150, and 300 mg/kg BW. Control‐SAT: satellite control treated with vehicle; XAE 300‐SAT: satellite of high dose of extract (300 mg/kg). Data are expressed as mean ± SEM (*n* = 5). Sge = *Stratum germinativum*; Sg = *Stratum granulosum*, Sc = *Stratum corneum*, Lv = vaginal lumen, Ch = chorion Lu = lumen; Sc = stratum corneum; SG = stratum granulosum; Sg = stratum germinativum, Ch = chorion, testis; Gc = germ cells; Se = seminiferous epithelium, It = interstitial tissue, Lst = lumen of seminiferous tube; epididymis; Ee = epididymal epithelium, Spz = spermatozoon; seminal vesicle; Ct = connective tissue; Es = eosinophilic secretion. Ductus deferens; Mu = muscularis, Dde = ductus deferens epithelium, L = lumen.

In males (Figure 5), the structure of the various organs in animals receiving XAE was similar to that in animals receiving distilled water (control). In fact, the cross‐sections of the testes, prostate, seminal vesicle, epididymis, and vas deferens of experimental rats show a normal microarchitecture. The structure of the testis shows the presence of seminiferous tubules with male sex cells at different stages of development, from the periphery to the lumen. On the prostate, glandular epithelium and eosinophilic secretions are visible, while the epididymis was marked by the presence of spermatozoa in the lumen of the epididymal tube and epididymal epithelium. As for the seminal vesicle, sections from normal animals showed a seminal epithelium and a lumen containing eosinophilic secretions.

## 4. Discussion

Evaluating the toxicity of a plant extract is required and regarded as a good attempt in better understanding their purity, dosage, suitable extraction solvent, and adverse effects for the use of herbal drugs [25]. Antioxidants, which are phytochemicals, play vital roles in human health [26] and against pathogenic infectious diseases. The antioxidant properties of XAE have been attributed to some phytochemical ingredients contained in them, as previously demonstrated by Nguedia et al. [16], which exhibit a wide range of biological effects [27].

In the acute toxicity investigation, variations in weekly body weight were insignificant between test and control groups. No fatalities or discernible signs of adverse effects, including alterations in behavior, were observed following a singular administration of XAE at a dose of 2000 mg/kg, followed by a 2‐week monitoring period. This implies that the lethal dose (LD_50_) of XAE surpasses 2000 mg/kg in rats, affirming its benign character [28]. Consequently, the extract falls within the nontoxic classification, aligning with Category 5 according to the Globally Harmonized Classification System for Chemical Substances and Mixtures (GHS) adopted by the OECD [23]. Moreover, LD_50_ values are conventionally ascertained for plants commonly utilized as food and/or medicinal herbs [29, 30].

The 90‐day subchronic toxicity investigation revealed no notable alterations in the weekly body weight variations between the treated and control groups, with no statistical differences observed in both male and female Wistar rats. Similarly, there were no significant changes detected in the relative organ weights of both sexes following oral administration of XAE for the 90‐day treatment period. Blood cells, being a type of connective tissue in mammals, play a crucial role in transporting oxygen, nutrients, and foreign particles to their designated destinations, including potentially toxic substances. Any impairment to these cells can indicate the physiological conditions, inflammations, and infections present in the animals [31]. The oral extract administration had a significant effect on some hematological values in the experimental animals. Furthermore, the extract shows no significant changes in RBCs, hematocrit, and Hb levels in all treated groups except XAE at 75 mg/kg BW in female rats and 300 mg/kg in male rats that significantly decreased the above parameters in comparison with their control. Abnormalities in nuclear maturation, identified by the presence of macrocytic RBCs with normal hemoglobin and MCHC levels, typically arise from a deficiency in vitamin B12 or folate [32]. This indicates the potential impact of the extract on hematopoiesis in the experimental animals and its ability to maintain the integrity of RBC membranes, potentially reversing anemia in Wistar rats [33] at significant doses. There were no notable fluctuations observed in the WBC counts among male and female rats. Nonetheless, a significant decrease in lymphocyte % was noted at the dose of 75 mg/kg BW in females and 300 mg/kg BW in male rats compared to the control group. Furthermore, an increase in granulocytes was observed in female rats across all tested dosages and in male rats at the dosage of 300 mg/kg BW. These findings corroborate earlier studies recognizing XAE as a promoter of packed cell volume (PCV), hemoglobin, WBC, and neutrophils in rats [34], validating the traditional application of the extract as a tonic and immune enhancer. The heightened neutrophil count may suggest a favorable response in bolstering the body’s overall resistance to infections [35]. The platelet levels significantly decreased in groups receiving XAE at all tested doses in female rats and in male rats at 300 mg/kg BW in comparison with their control group. Reduction in platelet count in experimental animals has been reported to indicate an adverse effect on the oxygen‐carrying capacity of the blood and may reflect impaired thrombopoiesis [27]. Thrombocytopenia, defined as a significant decrease in platelet count, carries the risk of impaired hemostasis, potentially increasing the tendency for bleeding including nasal, gingival, gastrointestinal, or in severe cases intracranial hemorrhage. This is a particular concern at the highest dose tested (300 mg/kg) and may limit the safety of long‐term use at this dose. Results from this study show that the platelet count was significantly (*p* ≤ 0.001) decreased at all tested doses in female rats and 300 mg/kg BW in the male Wistar rats (*p* ≤ 0.01), revealing that the oxygen‐carrying capacity of the blood and platelet production (thrombopoiesis) were adversely affected by XAE administration.

The kidneys and liver work in tandem to maintain internal balance, aiding in the removal of waste products and reabsorption of beneficial substances by the kidneys. Creatine, synthesized in the liver, is subsequently released into the bloodstream. In the process of creatine phosphate metabolism, creatinine, a waste byproduct, is produced and should be excreted by the kidneys. Elevated levels of creatinine and urea in the blood may indicate possible kidney dysfunction [36]. As plasma creatinine concentration typically remains constant, elevated levels suggest inadequate elimination linked to renal dysfunction [37]. Nevertheless, there were no notable alterations observed in creatinine and urea levels in both female and male subjects across all tested doses. The ingestion of XAE fruit extract did not lead to a significant rise in creatinine levels, suggesting an absence of nephrotoxicity under the study conditions.

The liver, a crucial organ for drug metabolism, plays a pivotal role in maintaining bodily equilibrium. Any abnormalities in hepatic cells can disrupt the normal detoxification process in animals [38, 39]. Aminotransferases (ALT and AST) are key serum markers of hepatic function, and their elevation in serum levels indicates liver damage and cellular injury [40]. XAE significantly reduced these parameters in both male and female rats at all tested doses, suggesting that the extract did not induce hepatocellular damage under the study conditions. These findings are consistent with an absence of extract‐induced hepatotoxicity, as supported by histopathological observations in Ateba et al. [29]. These were further reinforced by the absence of histopathological variations in the livers of animals treated with XAE compared to the control group. An escalation in total serum bilirubin levels is often linked with increased hemolysis [41] or conditions, such as primary biliary cirrhosis and hepatic veno‐occlusive disease (cholestasis), which may lead to extensive hepatocellular necrosis [42]. Total serum protein levels not only serve as a crude gauge of protein status but also reflect notable functional changes in kidney and liver functions. Elevated levels could indicate chronic inflammation or liver infections [43]. However, except for a noteworthy decrease (*p* < 0.05) observed at the dose of 300 mg/kg in male rats, no significant alterations were observed in protein and bilirubin levels in both male and female rats. This implies a potential liver‐protective effect of the extract, as supported by histopathological observations and the prevention of hemolysis.

An atherogenic lipid profile, characterized by elevated levels of TC, TG, low‐density lipoprotein (LDL), and low levels of HDL, is a primary risk factor for developing cardiovascular diseases [44, 45]. Consumption of XAE has been shown to reduce lipid composition and promote a favorable lipid profile in both serum and postmitochondrial fractions (PMF) of visceral organs in experimental hypercholesterolemia [46]. Accordingly, no significant changes were observed in these parameters in female and male rats after 90 days of treatment. Moreover, no significant alterations were noted in the satellite groups. These findings suggest that XAE can improve the lipid profile and potentially play a role in preventing cardiovascular events and arteriosclerosis.

Vital organs demonstrate a high susceptibility to harmful substances, and any resulting enlargement of these organs serves as a clear indication of toxicity, aiding in the assessment of toxicity levels, enzyme activation, physiological disruptions, and damage [4]. Following 90 days of oral ingestion of XAE extract in both male and female rats, histopathological analysis of the liver, kidneys, lungs, spleen, heart, brain, ovaries, and testes revealed no abnormalities or detrimental alterations at any dosage level compared to control rats.

## 5. Conclusion

The administration of a single dose of 2000 mg/kg of XAE dry fruit ethanol extract did not result in any observed signs of toxicity or mortality upon ingestion. Furthermore, its 90‐day oral administration did not affect the body weight or relative weights of organs in either sex and biochemically assessed parameters. Additionally, it induced no significant changes in hepatic (ALT, AST, and bilirubin) or renal (creatinine and urea) biomarkers in both male and female rats. However, the significant decrease in platelet levels observed suggests that prolonged use of XAE may have adverse effects on hemostasis, increasing the risk of impaired blood clotting and anemia, particularly at the 300 mg/kg dose. These findings are of particular concern given the traditional use of this plant during late pregnancy and the postpartum period, where thrombocytopenia could exacerbate the risk of hemorrhage. Further studies may be warranted to investigate the chronic (180 days) effects of the extract, including identifying potential causative agents and elucidating the mechanisms by which any potential damages may be remediated.

**Nomenclature**
ALT: alanine transaminase
ANOVA: analysis of variance
AST: aspartate transaminase
GHS: Chemical Substances and Mixtures
Hb: hemoglobin
HDL‐C: high‐density lipoprotein
LD_50_
: lethal dose 50
MCH: mean corpuscular hemoglobin
MCHC: mean corpuscular hemoglobin concentration
MCV: mean corpuscular volume
OECD: Organization for Economic Cooperation and Development
PCV: packed cell volume
PMF: postmitochondrial fractions
RBCs: red blood cell count
SAT: satellite
SEM: standard error of the mean
TC: total cholesterol
WBCs: total white blood cell count
TG: triglycerides
XAE: *Xylopia aethiopica*

## Author Contributions

SZ and ND designed the study. MYN, OBKN, and BM performed the in vivo experiment. HHAN and MYN drafted the manuscript.

## Funding

The authors have nothing to report.

## Disclosure

All authors have revised and approved the final manuscript.

## Ethics Statement

Housing and animal treatments were approved by the Faculty of Science’s Joint Institutional Review Board Animal and Human Bioethics (Ref: BTC‐JIRB2021‐010), which followed the European Union’s rules on both human and animal welfare (EEC Council 86/609).

## Consent

The authors have nothing to report.

## Conflicts of Interest

The authors declare no conflicts of interest.

## Supporting Information

Additional supporting information can be found online in the Supporting Information section.

## Supporting information


**Supporting Information** Supporting data 1. Signs of toxicity and general behavior of female rats. During the 14‐day observation, post‐exposition period to a bolus of 2000 mg/kg of ethanol dry fruit extract of XAE, there were no signs of fatalities or indications of toxicity noted in the behavioral assessments (including mobility, sensitivity to noise, fur condition, grooming behavior, and aggressiveness) of the treated rats.

## Data Availability

The data that support the findings of this study are available from the corresponding author upon reasonable request.
