# Supplementary material for: Acute (14‐Day) and Subchronic (90‐Day) Toxicity Evaluation of the Dried Fruit Spice Xylopia aethiopica (Dunal) A. Rich. (Annonaceae) in Male and Female Wistar Rats
Source: J Toxicol. 2026 May 21;2026:2002718. doi: 10.1155/jt/2002718 (PMC13195186; doi:10.1155/jt/2002718)
Supplement: Supplementary file 1 — Supporting Information Supporting data 1. Signs of toxicity and general behavior of female rats. During the 14‐day observation, post‐exposition period to a bolus of 2000 mg/kg of ethanol dry fruit extract of XAE, there were no signs of fatalities or indications of toxicity noted in the behavioral assessments (including mobility, sensitivity to noise, fur condition, grooming behavior, and aggressiveness) of the treated rats. [file JT-2026-2002718-s001.docx]

**Suppl data 1**. Signs of toxicity and general behavior of female rats

| **Time** | **30 min** | | **2 h** | | **4 h** | | **24 h** | | **48 h** | | **Week 1** | | **Week 2** | |
| --- | --- | --- | --- | --- | --- | --- | --- | --- | --- | --- | --- | --- | --- | --- |
| Observation | C | XAE | C | XAE | C | XAE | C | XAE | C | XAE | C | XAE | C | XAE |
| Fur | N | N | N | N | N | N | N | N | N | N | N | N | N | N |
| Sensitivity to noise | N | N | N | N | N | N | N | N | N | N | N | N | N | N |
| Mobility | N | N | N | N | N | N | N | N | N | N | N | N | N | N |
| Grooming | N | N | N | N | N | N | N | N | N | N | N | N | N | N |
| State of feces | N | N | N | N | N | N | N | N | N | N | N | N | N | N |
| Aggressivity | N | N+ | N | N+ | N | N | N | N | N | N | N | N | N | N |
| Dead | A | A | A | A | A | A | A | A | A | A | A | A | A | A |
| Tumor | A | A | A | A | A | A | A | A | A | A | A | A | A | A |

C = Control rats receiving distilled water; XAE = rat treated with *X. aethiopica* ethanol dried fruits at 2000 mg/kg; N = normal; A = absent; min = minute; h = hour.
